# Oneida County Veterinary Medical Association

**Published:** 1898-03

**Authors:** 


### ONEIDA COUNTY VETERINARY MEDICAL ASSOCIATION.

THE regular quarterly meeting was held at Stanwix Hall, on Tuesday afternoon, February 8, 1898. In the absence of President Morrow, of Onieda, Vice-President Huff, of Rome, occupied the chair. The other members present were: Dr. Stebbins, of West Winfield; Dr. Markham, of Port Leyden; Dr. Findlay, of Camden; Dr. Bettinger, of Canastota; and Dr. Currie, of Rome.

The committee appointed to obtain certificates of membership reported progress, though the certificates are not yet ready.

Dr. J. M. Currie, the essayist of the day, read an interesting paper on the subject of "Abortions in Cows," which he said was one of the most serious scourges that stock-breeders have to contend with, and he thought veterinarians should give the matter continuous attention, in order to investigate the means by which it might be averted. Among the causes named were foot-and-mouth disease, ergotted grasses, accidental diseases, tuberculosis; but the most common cause is, doubtless, infection. Among the preventive measures named were free accesss to salt or feeding on saline herbage. In closing, the speaker related some of his own experiences in such cases. A general discussion of the paper followed. The use of disinfectants, local applications, and internal antiseptics was recommended. Dr. Currie prophesied that in a few years some scientist would discover a serum to prevent it.

Dr. Huff also read a valuable paper on the same subject, confining himself to sporadic cases. The causes, he said, were numerous. Among them were accidents of various kinds. Sympathy is a possible cause, or a diseased condition of the system. He said, in conclusion, that he had been collecting notes on the subject since the last meeting, and decided to lay them before the members.

After some discussion of the paper, branching out into a talk on the results of feeding ensilage and sugar-beet pulp, Dr. Huff read a paper on "Bacteriology," of which he said no fewer than five hundred microörganisms have been isolated and described. Most of them are perfectly harmless, and some are positively healthful. While the science of bacteriology is of recent origin, as long ago as the sixteenth century a physician became convinced that some diseases were caused by living contagion.

At the close of Dr. Huff's paper, which was heard with close attention, the meeting adjourned.

